# Psychological trauma and evidence for enhanced vulnerability for posttraumatic stress disorder through previous trauma among West Nile refugees

**DOI:** 10.1186/1471-244X-4-34

**Published:** 2004-10-25

**Authors:** Frank Neuner, Maggie Schauer, Unni Karunakara, Christine Klaschik, Christina Robert, Thomas Elbert

**Affiliations:** 1 Department of Psychology, University of Konstanz and Center for Psychiatry Reichenau, D-78457 Konstanz, Germany; 2 vivo, Casella Postale no.17, Castelplanio Stazione, I-60032 Ancona, Italy; 3 Médecins sans Frontières, PO Box 10014, 1001 EA Amsterdam, The Netherlands; 4 Department of Family Social Science, University of Minnesota, St. Paul, MN 55108, USA

## Abstract

**Background:**

Political instability and the civil war in Southern Sudan have resulted in numerous atrocities, mass violence, and forced migration for vast parts of the civilian population in the West Nile region. High exposure to traumatic experiences has been particularly prominent in the Ugandan and Sudanese of the West Nile Region, representing an indication of the psychological strain posed by years of armed conflict.

**Methods:**

In this study the impact of traumatic events on the prevalence and severity of posttraumatic stress disorder (PTSD) in a random sample of 3.339 Ugandan nationals, Sudanese nationals, and Sudanese refugees (1.831 households) of the West Nile region is assessed.

**Results:**

Results show a positive correlation between the number of traumatic events and the number of endorsed PTSD symptoms. Of the 58 respondents who experienced the greatest number of traumatizing experiences, all reported symptoms which met the DSM-IV criteria for PTSD.

**Conclusions:**

There is a clear dose-effect relationship between traumatic exposure and PTSD in the studied populations with high levels of traumatic events. In this context, it is probable that any individual could develop PTSD regardless of other risk-factors once the trauma load reaches a certain threshold.

## Background

The debate about the impact of traumatic life events on psychiatric disorders has a long tradition in psychiatry. The introduction of posttraumatic stress disorder (PTSD) into the Diagnostic and Statistical Manual of Mental Disorders (DSM-III [1]) manifested the general recognition that a chronic condition consisting of characteristic symptoms including involuntary intrusions of the past, avoidance behavior and a condition of general hyperarrousal can be caused by traumatic exposure and must be viewed as mental disorder. Consequently, the original conceptualization of PTSD was based on the implicit assumption that the traumatic event is the main agent for the development of PTSD [2]. The initial idea was that traumatic events could cause PTSD in anyone regardless of pre-trauma vulnerability.

Contrary to this assumption, the following research showed that the development of chronic PTSD is rather the exception than the rule after the experience of a traumatic event. Community studies in the US showed that whereas more than 50% of the population reported the experience of a traumatic event, the prevalence of PTSD was not higher than 7.8% [3]. Among the different events studied, rape seemed to be the most adverse experience, with about 50% of victims developing chronic PTSD. But even studies that researched PTSD in those who experienced events considered to be most adverse, like torture in prison, found PTSD prevalence rates under 50% [4]. The realization that traumatic exposure is not a sufficient determinant of PTSD has stimulated vast research into risk and protection factors for the development of PTSD [5,6]. These studies show that pre-trauma developmental vulnerability (adverse childhood, psychiatric history, etc.), peri-traumatic factors (like peri-traumatic dissociation) [6], posttraumatic factors (like social support) as well as genetic factors [7], mediate the development of PTSD, although effect sizes were generally small.

A popular and intuitively plausible assumption in this context is the dose-response model of PTSD. This hypothesis predicts that the probability for the development of PTSD after the experience of a traumatic event mainly depends on the severity of trauma exposure. Some studies tried to test this hypothesis by relating the objective severity of the traumatic event to symptoms of PTSD. However, the empirical evidence for this model is scarce, with some findings supporting this hypothesis but many failing to confirm a relationship with meaningful effect sizes [8,9].

The probability of detecting a relationship between trauma exposure and PTSD depends on the range and variance of traumatic exposure that is present in the population studied. Studies investigating the relationship between the objective severity of single events and PTSD are restricted to a narrow variance of traumatic exposure. Community studies that assess trauma exposure across different types of traumatic events should be more adequate to examine a dose-effect hypothesis. From a worldwide perspective, even community studies in industrialized countries are restricted to a relatively narrow range of trauma exposure. In contrast, community studies in civil populations affected by war enable the examination of a much wider range of traumatic exposure. These populations present a continuum of subjects ranging from individuals without any history of traumatic events to victims with a history of high numbers of severe events that are rarely to be found in communities without a history of war. Studying a community sample of Cambodian refugees who had fled the Pol Pot regime, Mollica [10] actually confirmed a clear linear relationship between the number of traumatic events and symptoms of PTSD and depression. Other studies with refugee populations are in line with this result [11-14]. These studies suggests a specification of the dose-response model; i.e., that it is not the severity of a single traumatic event that is linearly related to symptoms of PTSD, but the severity of previous cumulative trauma exposure.

Consequently, it can be hypothesized that each individual who has experienced or is experiencing traumatic events will develop PTSD after reaching a certain threshold of traumatic exposure. As this threshold is probably very high, a large number of subjects exposed to a large variance of traumatic events is necessary to test this hypothesis. We examined the dose-response relationship in the context of a large survey in the West-Nile regions of Sudan and Uganda. The study included Ugandan nationals with a quite peaceful development in the last decade, as well as Sudanese nationals living in the Southern Sudan war region and Sudanese refugees who had fled to Uganda. Among these groups we expected a sufficient variance of traumatic exposure to test for the specified dose-response hypothesis, including an adequate number of subjects who had to experience a series of extremely severe traumatic events. Cumulative trauma exposure was estimated by assessing the number of different *traumatic event types *experienced or witnessed so far. We considered this measurement to be more reliable than assessing the frequency of traumatic events as many survivors of civil wars reported countless exposures to specific traumatic events. To examine the impact of recent traumatic exposure, we also assessed the traumatic event types experienced or witnessed in the last year.

## Methods

As part of a study designed to better understand the impact of forced migration on fertility, mortality, violence and traumatic stress among Sudanese nationals living in southern Sudan and Ugandan nationals and Sudanese refugees living in northern Uganda, we interviewed 3371 individuals from 1842 households in the Ugandan and Sudanese populations in the West Nile. Interviews were structured and were administered in the native languages of Lugbara or Juba Arabic. The study's design involved a multi-stage sampling design.

The full training of the interviewers took two months. The project objectives and the rationale behind the structure of the survey instrument as well as that of each question in the questionnaire were discussed in detail. Great attention was also paid to issues such as initial contacts, maintaining a professional attitude while in the field, avoiding influencing the respondent, and reducing interviewer and courtesy biases. The importance of collecting information by means of standardized questions so that the same question was asked to all respondents is stressed and questioning and probing skills were developed. Supervisors were instructed separately on data collection guidelines, their roles and their responsibility to ensure data quality. Keeping in mind the sensitive nature of some of the questions regarding violence and trauma and the fact that the team members were from the study population and probably had experiences similar to the respondents, a workshop on sexual and gender-based-violence was conducted by a consultant to the UNICEF office in Kampala, before the survey. The aim of this workshop was to increase awareness and sensitivity of the team towards respondents and their experiences. Another consultant to the project reviewed the team's interviewing skills and the project's data quality control measures just before the start of the survey. Problem areas were identified and remedied.

Data were complete and analyzed for N = 3179 respondents: 2,540 (75 %) of the respondents were women (15–50 years of age) and 831 (25%) were men (20–55 years of age). Details of the sampling, translation and assessment procedures, as well as the socio-demographic characteristics of the populations, have been described elsewhere [15].

Traumatic events were assessed using a checklist consisting of possible war and non-war related traumatic event types (i.e. witnessing or experiencing injury by a weapon or gun, beatings/torture, harassment by armed personnel, robbery/extortion, imprisonment, poisoning, rape or sexual abuse, beatings, abduction, child marriage, forced prostitution/sexual slavery, forced circumcision, etc.). The checklist was compiled after interviews with key informants (security personnel, doctors, community leaders, women's representatives) and 30 respondents from all three populations about their personal history of stressful events. Following these interviews, the single events obtained in these studies were rated as being potentially traumatic by experts. The following pilot checklist was pre-tested among further 44 Ugandans and Sudanese in areas not selected for the survey and modified according to the suggestions of the respondents. A primary item analysis based on inter-item correlations led to the exclusion of some events that were obviously not directly related to traumatic histories, e.g. the experiencing of witchcraft. Events included 19 experienced events and 12 witnessed events. Respondents were asked for each event type if they had experienced or witnessed such an event *ever *(i.e., lifetime experience) and if it happened *in the past year*. PTSD in respondents was assessed using the Posttraumatic Stress Diagnostic Scale (PDS), modified for assessment by trained lay interviewers [16]. The PDS is a self-report measure widely-used in industrialized countries as a screening instrument for the diagnosis and severity of PTSD based on DSM-IV Criteria.

Confidentiality was assured and it was explained that researchers were not working for any UN or Ugandan government organization. Informed consent was obtained using a standardized form explaining the potential risks of participation and explaining that no compensation would be provided. Informed consent forms were signed by the respondent and a witness; fingerprints were taken from illiterate respondents. No financial incentives were provided and respondents were informed that no improvements in living conditions were to be expected as a result of participating in the survey. Respondents were provided with referrals to counseling services provided by NGOs where available.

## Results

**Figure 1:**
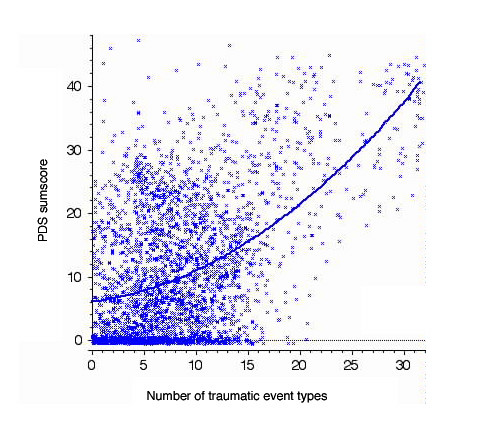
**Scatterplot of number of traumatic event types for whole life and severity of PTSD symptoms. **A number randomly chosen in the interval between -.05 and +0.5 was added to both the abscissa and the ordinate to visualize overlapping points.

As no major clustering effects were expected in this large sample, statistical analyses were carried out on unweighted data. To examine the relationship between continuous PTSD symptoms and the number of event types reported, we correlated the PDS score and its subscales, intrusion, avoidance and arousal with the number of event types. The number of event types in life correlated with the frequency of intrusions (r = .49), hyperarousal (r = .41) and avoidance (r = .47), all P < 0.001. The PDS sumscore correlated significantly (P < 0.001) with the number of traumatic events in the past year (r = .45) and for the whole life (r = .49; see figure1).

**Figure 2:**
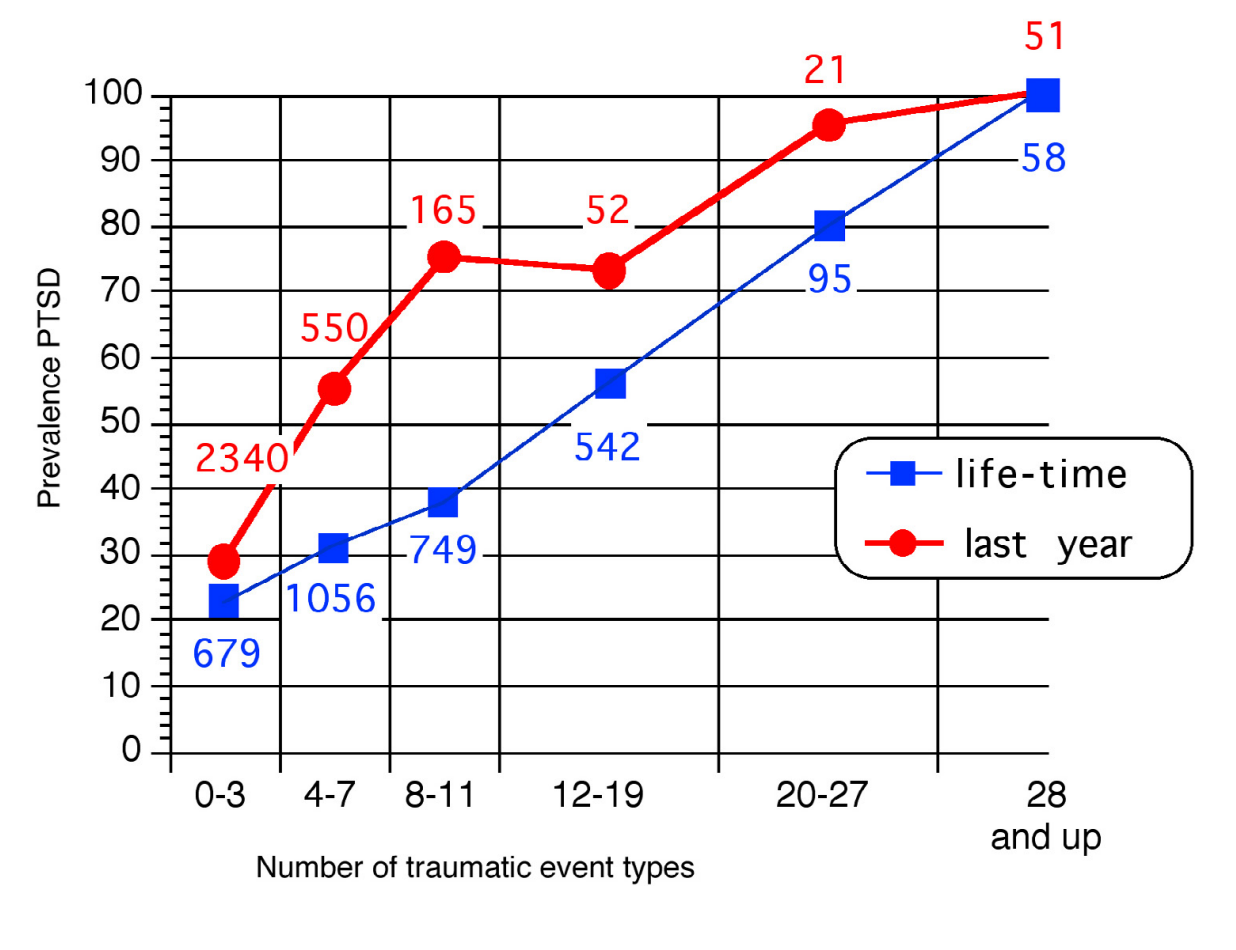
**Prevalence of PTSD and number of individuals in groups of respondents. **In this figure respondents are pooled on the basis of number of traumatic event types reported for whole life and last year.

Overall, 31.6% of the male and 40.1% of the female respondents (N = 3179) fulfilled the DSM criteria for a PTSD-diagnosis. We divided the whole population studied in the survey into different groups based on the number of traumatic event types reported, separately for the events reported for *last year *and *in life*. The initial division was made as follows: the first group consisted of respondents endorsing 0–3 event types, the second group consisted of individuals endorsing 4–7 event types. Each following group endorsed an additional four or more event types. Because the number of individuals in the groups of 12–15, 16–19, 20–23 and 24–27 event types was very small for the analyses of events reported last year (*n *= 38, 14, 8, 13, respectively), these groups were merged to two groups of 12–19 and 20–27 event types. Figure 2 shows the number of individuals and the prevalence of PTSD in these groups, separately for the groups based on the events reported for the whole life and for last year. The presentation indicates a near linear rise for increasing psychological strain with the number of traumatic event types ranging from 23% in respondents who reported three or fewer traumatizing experiences to 100% prevalence of PTSD in those who report 28 or more traumatic event types in their past. Figures related to traumatic event types in the past year display and even more pronounced increase of PTSD symptoms with significantly higher prevalence rates for the first three categories of numbers of events (Figure 2).

## Discussion

High prevalence rates of PTSD have been reported for three different population groups in the West Nile: Sudanese nationals (44.6%), Sudanese refugees (50.5%) and Ugandan residents (23.2%) [15]. Here we show that the exposure to traumatic events and the number of different types of traumatic experiences in particular can account for the different proportion of PTSD cases. The prediction of increased PTSD prevalence with increasing number of traumatic events is consistent with other studies investigating victims of organized violence [11-14].

As demonstrated, the number of traumatic events correlated equally strong with avoidance and with re-experiencing symptoms but coefficients were weaker, although still significant, for the hyperarousal cluster. These results are in agreement with [17], who also found a strong correlation between cumulative trauma and symptoms of re-experiencing and avoidance. Contrary to these findings, Mollica [10] could not find a correlation with avoidance symptoms. Problems in the translation of the avoidance items in the PTSD instruments might be responsible for this difference, as subtle modification in the translation process may turn PTSD avoidance criteria (like "less interest in important activities" or "feeling as if future plans will not come true") into unspecific depressive items that are unrelated to a traumatic experiences.

Typically, even severe single traumatic event produce PTSD in not more than half of those affected. Therefore, PTSD is not an inevitable consequence of potentially traumatizing events. Results from this study, however, suggest that there may be no ultimate resilience to ward off PTSD or that a psychobiological breaking point exists for even the most resistant individual. In the three population groups that were surveyed, each respondent experiencing 28 or more different traumatic event types developed the full set of symptoms of PTSD. This cumulative trauma threshold identified in this study is very high and affected only a small minority of persons even in a war-torn population. Nevertheless, if the cumulative exposure to traumatic events is high enough, these results indicate that anybody will develop chronic PTSD. We conclude that there is no ultimate resilience to traumatic stress and that the repeated occurrence of traumatic stress has a cumulative damaging effect on the mental health of the victim. In these conditions, the effect of pre-trauma factors is reduced to the modulation of the probability of exposure to traumatic events itself. The factors that determine who is exposed to many traumatic events and who manages to flee to secure places may depend on pre-trauma psychological factors. Further studies with war-populations should examine whether the exposure to traumatic events only depends on uncontrollable external factors or whether individual factors contribute to a person's ability to seek safe places.

## Conclusions

High levels of trauma exposure is found in populations affected by civil war. We show that PTSD, the major psychological consequence of war events, is linearly correlated with traumatic exposure, thus explaining the high prevalence rates of PTSD generally found in war-torn societies. These findings highlight the need for reducing the frequent exposure to traumatic events by preventing wars, controlling the violence in wars, and providing safe and stable living environments for refugees. At the same time, the presence of high numbers of PTSD cases requires the implementation of individual and community based treatment programs. Given very limited resources in refugee communities, these centers must be created to provide short-term care and must be manageable by local personnel [18,19]. The provision of appropriate mental health assistance is necessary to break the vicious cycle of violence and psychological morbidity.

## Competing interests

The author(s) declare that they have no competing interests.

## Authors' contributions

FN, MS, UK & TE designed the study. FN, MS and UK composed the set of instruments. UK was responsible for original instrument translation and data collection, FN, MS, CK and TE for the validation part. FN and CK performed the data analysis. CR, TE and FN drafted the original manuscript and all authors revised and approved the final manuscript.

## Pre-publication history

The pre-publication history for this paper can be accessed here:


